# Analysis of Fatty Acid Profile, α-Tocopherol, Squalene and Cholesterol Content in Edible Parts and By-Products of South Pacific Wild Fishes

**DOI:** 10.3390/md23030104

**Published:** 2025-02-27

**Authors:** Sussi López-Puebla, María Fernanda Arias-Santé, Jaime Romero, Adriano Costa de Camargo, Miguel Ángel Rincón-Cervera

**Affiliations:** 1 Institute of Nutrition and Food Technology, University of Chile, Macul, Santiago 7830490, Chile; sussi.lopez@inta.uchile.cl (S.L.-P.); ma.fernanda.arias@inta.uchile.cl (M.F.A.-S.); jromero@inta.uchile.cl (J.R.); adrianodecamargo@inta.uchile.cl (A.C.d.C.); 2 Food Technology Division, University of Almería, 04120 Almería, Spain

**Keywords:** fish, by-products, omega-3 fatty acids, tocopherol, squalene, cholesterol, nutritional lipid index

## Abstract

Fish are generally rich sources of n-3 polyunsaturated fatty acids such as EPA and DHA, and although the edible part (fillet) has been analyzed in many species, less is known about the composition of fish by-products. The analysis of these materials allows them to be evaluated as raw sources of EPA and DHA, thus contributing to sustainable practices to produce healthy oils for human consumption. This work provides information on the fatty acid profiles, lipid quality indices and α-tocopherol, squalene and cholesterol contents in fillets, heads, bones and viscera of three fish species (anchovy, chub mackerel and Chilean jack mackerel). Samples were lyophilized and lipids were extracted using either the Folch or Hara and Radin methods. FA profiles were obtained by gas chromatography coupled with flame ionization detection, and tocopherol, squalene and cholesterol analyses were performed by high performance liquid chromatography with UV-visible detection. The highest levels of EPA were found in anchovy fillet (18.9–20.6%) and bone (14.7%), while DHA was more abundant in anchovy fillet (16.9–22.0%) and Chilean jack mackerel fillet (15.4–16.6%) and bone (13.1–13.8%). α-Tocopherol, squalene and cholesterol contents ranged from 0.18 to 1.35 mg/100 g, 0.07 to 0.80 mg/100 g and 30.46 to 246.17 mg/100 g, respectively, in the different tissues analyzed from the three fish species.

## 1. Introduction

Marine resources play a fundamental role in the global food chain, and a notable increase in the consumption of seafood products has been noticed in recent years because they are widely recognized as an essential component of a balanced diet and a healthy lifestyle. Fish is a rich source of high-quality proteins, lipid-soluble vitamins, essential minerals and polyunsaturated fatty acids from the n-3 family (n-3 PUFA) [1,2]. In this regard, eicosapentaenoic acid (EPA, C20:5 n-3) and docosahexaenoic acid (DHA, C22:6 n-3) are the two most known n-3 PUFA due to their role in many physiological process in the human body such as fetal, neurological and visual development, maternal and child health, aging, cell signaling pathways and also because of their effect to prevent or alleviate several pathologies such as diabetes and cancer, as well as autoimmune, neurodegenerative and cardiovascular diseases [3,4,5,6]. EPA and DHA are also precursors of lipid mediators (eicosanoids and docosanoids, respectively) with a potent anti-inflammatory activity and contribute to improve lipid profile, blood pressure, platelet aggregation and vascular function [5,6]. EPA and DHA are considered conditionally essential n-3 PUFA because although they can be synthesized endogenously from their precursor α-linolenic acid (ALA, C18:3 n-3), the efficiency of this conversion by the human metabolism is low, and it is therefore recommended that EPA and DHA are provided directly through the diet [7].

Some of our previous works have highlighted that the fillets of some edible fish species consumed in Chile are rich sources of EPA and DHA, such as Peruvian morwong (*Chirodactylus variegatus*), Pacific sandperch (*Prolatilus jugularis*), yellowtail amberjack (*Seriola lalandi*), mackerel (*Scomber japonicus*) and Chilean jack mackerel (*Trachurus murphyi*) [8,9]. However, industrial and artisanal fishing activities generate wastes in the form of by-products, including non-commercial and non-edible parts and secondary species, and it has been estimated that approximately 50% of the fish body weight is generated as waste during the processing operations [10,11]. Currently, fish by-products are used to produce fishmeal, fertilizers and fish oil or they are directly discarded into the environment, thus raising questions about the efficiency and sustainability of fish waste management [10,11,12]. Nowadays, the use of fish by-products as an alternative source of EPA and DHA is receiving an increasing interest to minimize the current overexploitation situation of many fishing grounds and to meet the Sustainable Development Goals included in the 2030 Agenda.

Fish by-products such as trimmings, bones, head and viscera constitute a valuable source of nutritionally relevant lipids. For instance, it has been reported that the oils extracted from the head and gills of catfish (*Clarias macrocephalus*) and Atlantic mackerel (*Scomber scombrus*) contained 3–4% EPA and 5–9% DHA of total FA, respectively [12], whereas 9–12% EPA and 21–27% DHA were found in the oils extracted from the head, skin, and roes of Pacific blue mackerel (*Scomber australasicus*) [11]. Oils extracted from the head, viscera and bones of trout (*Oncorhynchus mykiss*) achieved levels of 8–9% EPA and 6–7% DHA [13].

The artisanal fishing port located in Coquimbo, Chile (−29.94378, −71.32307), is one of the main landing poles in the country, and large volumes of fish discards are produced as a result of fish processing [10]. The management of this waste is not efficient, leading to decomposition and dumping of the excess waste in the sea, which causes sanitary and environmental problems. The most commonly caught species include anchovy (*Engraulis ringens*), chub mackerel (*Scomber japonicus*) and Chilean jack mackerel (*Trachurus murphyi*), and these three species have been identified as the main contributors to the fish waste generated in the port [10].

Information on the composition of oils extracted from fish by-products is very scarce in Chile, and few studies have addressed the characterization of the lipid profile of edible fish consumed in the country. The aim of this work was to provide new data on the FA profiles and α-tocopherol, cholesterol and squalene contents of edible parts (fillets) and by-products (head, viscera and bones) of anchovy, chub mackerel and Chilean jack mackerel collected in Chile. Several lipid quality indices such as the atherogenic index (AI), thrombogenic index (TI), hypocholesterolemic/hypercholesterolemic FA ratio (HH) and fish lipid quality index (FLQ) were also calculated. In addition, two methods of lipid extraction were tested on all samples (the Folch method and an alternative extraction with a mixture of *n*-hexane and 2-propanol), and the results were compared in terms of lipid extraction yields and fatty acid profiles.

In addition to fatty acids, fish lipids contain other bioactive compounds such as tocopherols (vitamin E), squalene and cholesterol. Vitamin E is a fat-soluble antioxidant that may help prevent or alleviate cardiovascular and age-related conditions, including neurological disorders, and possesses anticancer and anti-inflammatory properties [14]. Squalene is a triterpenoid compound naturally available in animal and vegetal sources with cardioprotective, antibacterial, antioxidant and antitumor properties [15]. Regarding cholesterol, while it is a vital component for human cells, excess consumption has been associated with an increased risk of cardiovascular disease, and a recommended daily intake limit of 300 mg has been established to mitigate adverse cardiovascular effects [16]. Since cholesterol is predominantly found in animal tissues, it is important to evaluate the levels of this lipophilic compound in fish fillets.

Although it is known that fatty acid profiles, as well as the concentration of bioactive compounds of fish, may vary according to seasonal, environmental and biological factors, this study was designed as a cross-sectional study (each fish species studied was sampled at a single time point instead of sampling at multiple times during the fishing season) with the aim to specifically evaluate potential differences in fatty acid profiles, lipid quality indexes and the amount of α-tocopherol, squalene and cholesterol between edible and non-edible parts of three of the most commonly caught species in Chile: anchovy (*E. ringens*), chub mackerel (*S. japonicus*) and Chilean jack mackerel (*T. murphyi*).

The current work provides useful data for the potential valorization of fish processing wastes as sources of EPA, DHA and other important lipophilic compounds to meet the growing demand for marine-derived foods and supplements for animal and human consumption.

## 2. Results

### 2.1. Moisture and Lipid Content in Fish Fillets and By-Products

Moisture values ranged between 62.0% in chub mackerel fillet and 76.0% in anchovy fillet (Table 1). The bones showed lower moisture values than the rest of the analyzed tissues in anchovy and Chilean jack mackerel, although moisture was even lower in the fillet in the case of chub mackerel. The average moisture percentages considering the four studied tissues were 72.6, 69.4 and 65.9% for anchovy, Chilean jack mackerel and chub mackerel, respectively.

Regarding lipid yields using the Folch method or the mixture of *n*-hexane and 2-propanol, no significant differences were found in most tissues except in the head and fillet of anchovy, the viscera of Chilean jack mackerel and the head of chub mackerel, where significantly higher yields were found using the Folch extraction (Table 1). In anchovy, the highest lipid content was found in the viscera (25.6 g/100 g dw), followed by the head (17.0 g/100 g dw) and bones (14.5 g/100 g dw), whereas the lowest lipid content was found in the fillet (9.6 g/100 g dw). In Chilean jack mackerel, the viscera and head were the tissues with the highest lipid content, followed by bones and fillet. Chub mackerel was the species with the highest lipid amount in all tissues compared with their counterparts of anchovy and Chilean jack mackerel, the viscera of chub mackerel being the tissue with the highest lipid content (45.8 g/100 g dw) among all analyzed samples in this study.

### 2.2. Fatty Acid Profiles

The fatty acid profiles of fillet, head, bones and viscera of anchovy are reported in Table 2. The viscera was the tissue with the highest proportion of saturated FA (SFA) (46.76–49.62% of total FA), followed by the head (41.87–43.71%), bones (39.97–40.66%), and fillet (32.77–33.24%). The Folch extraction gave significantly higher values of SFAs than the *n*-hexane:2-propanol extraction only in the viscera, whereas the proportions were not significantly different in the other assayed tissues. The main SFA was palmitic acid (PA, C16:0) in all cases, followed by myristic acid (MA, C14:0) and stearic acid (SA, C18:0). Regarding total monounsaturated FAs (MUFAs), significantly lower values were found in fillets (18.39–21.10%) compared to the by-products, which showed similar values of total MUFAs ranging from 26.73% to 28.86% of total FA. The most abundant MUFAs were palmitoleic acid (PaA, C16:1 n-7) and oleic acid (OA, C18:1 n-9) in all analyzed samples. The extraction with *n*-hexane:2-propanol led to significantly higher values of total MUFAs in the fillet and viscera, whereas no significant differences were found in the head and bones. Contrarily to SFAs and MUFAs, values of total PUFAs were significantly higher in anchovy fillets (46.12–48.37%) than in the bones (31.43–32.19%), head (27.43–30.39%) and viscera (22.28–26.50%). EPA and DHA were the most abundant PUFAs in all studied samples, fillet being the tissue with the highest proportions of both n-3 PUFAs. The Folch extraction led to significantly higher DHA values in the fillet (22.05%) and viscera (6.30%) than the extraction with *n*-hexane:2-propanol (16.87 and 4.92%, respectively) with no significant differences in the head and bones. The same fact was observed for EPA except in anchovy fillet, where the EPA proportion was significantly higher in the lipids extracted with *n*-hexane:2-propanol (20.56%) than in those obtained with the Folch extraction (18.88%). The proportion of DHA in anchovy tissues was generally lower than that of EPA, except in the lipids extracted from fillets with the Folch procedure.

The fatty acid profiles of fillets and by-products of Chilean jack mackerel are shown in Table 3. Values of total SFA were significantly higher in the viscera (49.58–51.54%) than in the rest of the analyzed tissues, with no significant differences among them and values ranging from 35.68 to 37.83%. PA was the most abundant SFA in all cases, followed by SA. The proportion of total MUFA was significantly higher in the viscera than in the rest of the tissues, and different values were found by using the Folch extraction (36.99%) or the combination of *n*-hexane and 2-propanol (45.50%). Contrary to what was observed in anchovy, the main MUFA found in Chilean jack mackerel tissues was OA, which was particularly abundant in the viscera (22.13–30.34% depending on the lipid extraction method) and remained around 20% in the rest of the analyzed samples of Chilean jack mackerel. Regarding total PUFAs, significantly lower values were found in the viscera compared to the other tissues, and the viscera lipids extracted with the Folch method showed a significantly higher PUFA proportion (11.48%) compared to the lipids extracted with *n*-hexane and 2-propanol (4.92%). DHA was the most abundant PUFA in fillets and bones, whereas values were very similar to those of EPA in the head and viscera.

The fatty acid profiles of fillets and by-products of chub mackerel are reported in Table 4. The proportion of total SFAs was significantly higher in the head (47.53–47.78% of all FAs) followed by the viscera (45.04–45.14%), whereas the fillet and bones showed lower values for SFAs (35.77–37.13%). PA was the main SFA in all tissues, with higher values in the head (30.4%) and viscera (30.3%) than in the bones (23.9%) and fillet (24.9%), and there were no significant differences regarding the lipid extraction method. Total MUFAs were significantly more abundant in the viscera than in the other samples of chub mackerel, and no differences were found regarding the lipid extraction method. OA showed the highest values among all MUFAs, whereas eicosenoic acid (EO, C20:1 n-9) was found in much higher proportion than in anchovy and Chilean jack mackerel, reaching up to 8.0% of total FAs in the viscera and between 3.7 and 6.1% in the other analyzed tissues. Regarding total PUFAs, significantly lower values were found in the viscera (9.7–10.4%) than in the other tissues: head (13.0–14.5%), fillet (27.0–27.2%) and bones (28.9–29.6%), and no significant differences were found between lipid extraction methods. EPA and DHA showed similar values in each analyzed chub mackerel tissue, the bones (9.5–10.0% EPA, 10.9–11.9% DHA) and fillet (8.3–9.3% EPA, 9.9–10.8% DHA) being the samples with the highest proportions, followed by the head (3.5–4.0% EPA, 3.9–4.9% DHA) and viscera (3.1–3.2% EPA, 2.4–2.5% DHA).

### 2.3. Nutritional Quality Indices

The lipid quality indices of anchovy fillets and by-products are reported in Table 2. The n-6/n-3 PUFA ratio was lower in the fillet (0.07–0.08) and bones (0.14–0.16) than in the head (0.23–0.24) and viscera (0.23–0.29). The AI ranged from 0.74–0.76 in fillet to 1.44–1.61 in viscera, whereas the values for TI were between 0.20 and 0.21 in the fillet and 0.53 and 0.67 in the viscera. The opposite was observed for the HH and FLQ indices, with the highest values found in the fillets (2.04–2.08 for HH and 37.43–40.93 for FLQ) and the lowest in the viscera (0.88–1.03 for HH and 14.00–17.60 for FLQ). Intermediate values were observed in the head (1.17–1.26 for HH and 18.50–19.77 for FLQ) and the bones (1.31–1.35 for HH and 22.53–22.97 for FLQ).

The lipid quality indices of Chilean jack mackerel fillets and by-products are shown in Table 3. Very similar values for the n-6/n-3 PUFA ratio were observed in all analyzed tissues (0.11–0.14) except in the viscera, where this index increased to 0.31–0.35. The same trend was found for AI (0.65–0.75 in the fillet, head, and bones and 1.04–1.29 in viscera) and TI (0.29–0.33 in all tissues but the viscera, rising to 1.01–1.30). HH moved in a narrow interval in the fillet, head and bones (1.84–2.01) and fell to 0.92–1.06 in the viscera, which was also noticed for FLQ (23.04–26.63 in all tissues except in the viscera, where it ranged from 2.47 to 6.84).

The fillets and bones of chub mackerel showed comparable values of all lipid quality indices with lower values than the head and viscera for the n-6/n-3 PUFA ratio (0.12–0.14), AI (0.67–0.68) and TI (0.33–0.36) and higher values for HH (1.78–1.88) and FLQ (19.17–21.35) (Table 4). The head and viscera showed similar values between them for the n-6/n-3 PUFA index (0.20–0.26), AI (0.96–1.12), TI (0.73–0.84) and HH (1.10–1.15). However, FLQ was increased more in the head (7.37–8.92) than in the viscera (5.61–5.70).

### 2.4. α-Tocopherol, Squalene and Cholesterol Content

**Figure 1:**
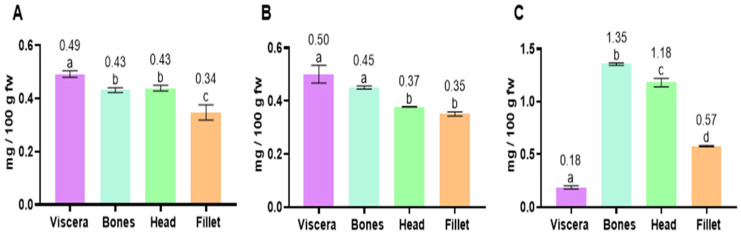
Contents of α-tocopherol (mg/100 g fw) in fish tissues (viscera, bones, head and fillet) of anchovy (**A**), Chilean jack mackerel (**B**) and chub mackerel (**C**). Results are shown as mean ± standard deviation (*n* = 3). Different letters in each column mean significantly different values (*p* < 0.05).

The α-tocopherol content in the tissues of the three fish species analyzed are shown in Figure 1. In anchovy, the amount of α-tocopherol was 0.34 and 0.49 mg/100 g fw in the fillet and viscera, respectively, with no significant difference between α-tocopherol content in the bones and head (0.43 mg/100 g) (Figure 1A). In Chilean jack mackerel, values were 0.35 and 0.50 mg/100 g in the fillet and viscera, respectively, whereas the bones and head showed intermediate amounts (0.45 and 0.38 mg/100 g, respectively) (Figure 1B). Regarding chub mackerel, significantly higher values of α-tocopherol were found in the bones (1.35 mg/100 g) and head (1.18 mg/100 g), whereas lower amounts were observed in the fillet (0.57 mg/100 g) and viscera (0.18 mg/100 g) (Figure 1C).

**Figure 2:**
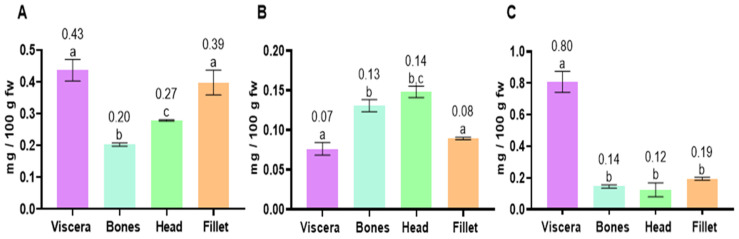
Contents of squalene (mg/100 g fw) in fish tissues (viscera, bones, head and fillet) of anchovy (**A**), Chilean jack mackerel (**B**) and chub mackerel (**C**). Results are shown as mean ± standard deviation (*n* = 3). Different letters in each column mean significantly different values (*p* < 0.05).

The squalene content in anchovy was significantly higher in the viscera (0.43 mg/100 g fw) and fillet (0.39 mg/100 g) than in the head (0.27 mg/100 g) and bones (0.20 mg/100 g) (Figure 2A), whereas in Chilean jack mackerel, the opposite trend was found, with significantly higher squalene amounts in the head and bones (0.14 and 0.13 mg/100 g, respectively) than in the fillet (0.08 mg/100 g) and viscera (0.07 mg/100 g) (Figure 2B). In chub mackerel, the viscera showed a much higher squalene amount (0.80 mg/100 g) than the rest of the analyzed tissues: the fillet (0.19 mg/100 g), bones (0.14 mg/100 g) and head (0.12 mg/100 g) (Figure 2C).

**Figure 3:**
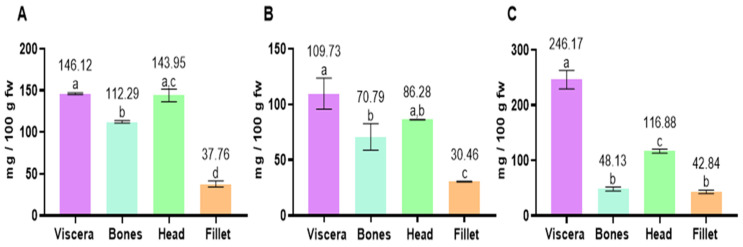
Contents of cholesterol (mg/100 g fw) in fish tissues (viscera, bones, head and fillet) of anchovy (**A**), Chilean jack mackerel (**B**) and chub mackerel (**C**). Results are shown as mean ± standard deviation (*n* = 3). Different letters in each column mean significantly different values (*p* < 0.05).

The lowest amounts of cholesterol were found in fish fillets in the three analyzed species, followed by the bones, head and viscera (Figure 3). Cholesterol contents in the fillets ranged from 30.46 mg/100 g fw in Chilean jack mackerel to 42.84 mg/100 g fw in chub mackerel. In the bones, the amount ranged from 48.13 to 112.29 mg/100 g fw in chub mackerel and anchovy, respectively. Regarding the head, cholesterol values were 86.28 and 143.95 mg/100 g fw in Chilean jack mackerel and anchovy, respectively. Furthermore, in the viscera, chub mackerel showed the highest amount of cholesterol (246.17 mg/100 g), followed by anchovy (146.12 mg/100 g) and Chilean jack mackerel (109.73 mg/100 g).

## 3. Discussion

### 3.1. Moisture and Lipid Content in Fish Fillets and By-Products

The moisture percentages found in this study are close to those previously reported in the literature for fish tissues [2]. Several authors have reported a moisture proportion of 75% in the fillet of Chilean jack mackerel (*Trachurus murphyi*) and values even higher than 80% in fillets of jack mackerel (*Trachurus trachurus*) collected on the Portuguese coast [17,18,19]. Other work found 80, 83, 83 and 73% moisture in the fillet, viscera, skin and bones of Indian jack mackerel (*Magalaspis cordyla*), respectively [20,21,22,23]. The moisture values for anchovy caught in the Black or Mediterranean Sea did not exceed 70% in fillets or the whole fish [21,22,23]. Regarding mackerel fillet, moisture values between 70 and 76% were reported in fishes caught in the Mediterranean Sea (Turkey and Tunisia) and in Atlantic mackerel caught in Portugal [24,25,26,27]. Moisture values of 45, 50 and 67% were found in blue mackerel (*Scomber australasicus*) head, skin and gonads, respectively, sampling fishes provided by a commercial fish processor [11].

Regarding the lipid extraction yield, no significant differences were found between the two extraction alternatives (Folch and *n*-hexane/2-propanol) in most cases, although slightly higher values were generally obtained with the Folch method, especially for anchovy head and fillet, Chilean jack mackerel viscera and chub mackerel head, where the Folch yield was significantly higher than that obtained with *n*-hexane/2-propanol. The Folch extraction is considered as a reference method for lipid extraction from animal tissues, but the use of chloroform raises several safety and environmental concerns due to its high hazard risk, and therefore, several alternatives have been tested to attempt lipid extraction with reasonably high yields [28]. The use of the less toxic and cheaper mixture of *n*-hexane and 2-propanol was proposed by Hara and Radin [29], and it was found that this technique was almost as efficient as the Folch and Soxhlet methods for lipid extraction from fish tissues [30], which is in agreement with the results obtained in the current study. Other techniques have also been widely used for lipid extraction from fish tissues, like Soxhlet and Bligh and Dyer, due to its efficiency and relatively simple handling [21,22,25]. However, the use of organic solvents combined with higher temperatures and longer operating times in Soxhlet processing make it advisable to look for safer and more cost-effective alternatives for lipid extraction [28].

Although fish by-products are not intended for direct consumption, as is the case with fillets, they could be good sources of healthy fatty acids and bioactive compounds for nutritional, nutraceutical or pharmaceutical purposes. All by-products studied in this work showed lipid amounts above 11.8 g/100 g dw, the viscera being the tissue with the significantly highest content in anchovy and chub mackerel and with a similar content to the head in Chilean jack mackerel. Other authors also found a higher lipid content in the viscera of fresh anchovy (*Engraulis encrasicholus*) from the Black Sea in Turkey (23.9%) than in the frame (15.5%), fillet (12.5%) and head (10.0%) [21]. Osako et al. analyzed the fillet, liver and stomach of blue mackerel (*Scomber australasicus*) caught in China, comparing different years of capture. The best performance was observed in the liver (9.4 to 11.8 g/100 g fw) with no major differences between the fillet and stomach (1.3 to 5.3 g/100 g fw, 1.1 to 1.3 g/100 g fw, respectively) [31]. Another study screened different subparts of the jack mackerel (*Magalaspis cordyla*) caught in India and found a better lipid yield in the spine (3 g/100 g fw) than in the viscera (2.5 g/100 g fw) and skin (2.0 g/100 g fw), with the lowest yield in the fillet (1.4 g/100 g fw) [20].

The lipid content of fish is highly dynamic and depends on factors such as the seasonal variations, food availability and water temperature, among others [32]. In addition, lipid extraction yields can vary regarding the processing and extraction methodology, and although the Folch extraction is the reference method for lipid extraction with analytical purposes, the use of “greener” alternatives involving environmentally safer and non-chlorinated solvents, as well as emerging technologies such as microwave-assisted extraction, ultrasound-assisted extraction, high hydrostatic pressures or fermentative extraction, is advisable when the lipid fractions of fish by-products are recovered at pilot or industrial scale for nutritional or nutraceutical purposes [33,34].

### 3.2. Fatty Acid Profile and Lipid Quality Indices

Fillets are the commonly edible part of fish, and the study of their FA profile is relevant from a nutritional view because fish is currently the main dietary source of EPA and DHA. The anchovy fillets analyzed in this work showed high proportions of both n-3 PUFAs and total PUFAs, and although lower values for EPA, DHA and total PUFAs were found in the fillets of Chilean jack mackerel and chub mackerel, they can be still considered good sources of these FAs. Previous studies have reported similar FA proportions in anchovy to those found in the current work. For example, Bayrakli found 34.3–35.2% SFAs, 23.2–23.8% MUFAs and 41.0–42.5% PUFAs in anchovy oil (*Engraulis encrasicolus*) extracted from the whole fish in different factories, with EPA and DHA values ranging from 9.3 to 9.6% and 13.5 to 15.9% of total FAs, respectively [35]. Di Bella et al. found 37.5% SFAs, 18.3% MUFAs and 44.2% PUFAs in fillets of anchovy (*E. encrasicolus*) from Italy, with values of 11.4% EPA and 22.1% DHA [36]. Park et al. analyzed the FA profiles of anchovy (*E. japonica*) fillets from South Korea and reported values of 43.6% SFAs, 22.7% MUFAs and 33.7% PUFAs, with a similar proportion of DHA (19.5%) and a lower proportion of EPA (12.7%) compared with the current study [37]. In general, the previously reported EPA values in anchovy fillets are lower than those found in this work, which may be related to the variability of FA profiles regarding fish species, catching season, or fish feeding, among others.

Previous studies have reported FA profiles of fillets of jack mackerel and related species. Merdzhanova et al. found 39.4% SFAs, 26.1% MUFAs and 35.0% PUFAs in skinned fillets of Mediterranean horse mackerel (*Trachurus mediterraneus*) sampled in a Bulgarian market, with values of 1.4% and 21.6% for EPA and DHA, respectively [38]. The fillets of horse mackerel (*Trachurus trachurus*) captured in Italy showed values of 33.1–37.6% SFAs, 19.6–24.5% MUFAs and 32.9–42.1% PUFAs, together with 5.3–6.4% EPA and 20.9–27.7% DHA [39], and another study found proportions of 37.8–44.4% SFAs, 22.4–23.9% MUFAs and 20.1–24.9% PUFAs (comprising 4.9–5.4% EPA and 11.1–15.0% DHA) for the same fish species captured in Turkey in different seasons [24]. In this line, the flesh of horse mackerel (*Trachurus trachurus*) caught off the Portuguese coast showed values of 26.9–30.5% SFAs, 23.8–32.4% MUFAs and 34.6–43.4% PUFAs, with 6.0–11.6% EPA and 15.2–24.5% DHA), with significant variations depending on the fishing season [17]. In general, DHA levels were found to be higher than EPA levels in the flesh of fish of the genus *Trachurus*, which is consistent with the results obtained in the present study.

Regarding the FA profiles of fillets of mackerel species, other authors have reported much higher levels of DHA than EPA, with wide variations depending on the fishing season and geographical location. For example, EPA levels in fillets of *Scomber scombrus* caught in Tunisia, *Scomber colias* caught in Portugal and *Scomberomorus maculatus* captured in the Gulf of Mexico were 4.6–7.8%, 6.8–16.7% and 3.3–9.3%, respectively, whereas DHA levels were 35.7–40.1%, 13.8–48.6% and 9.1–40.3% for the same species [25,26,40]. Osako et al. found EPA values between 5.1 and 8.1% and DHA values between 18.6 and 27.5% in the fillet of *Scomber australasicus* caught off the China Sea in different seasons [29]. In one of our previous works, considerably higher values of DHA than EPA were found in the fillet of the same chub mackerel species (*Scomber japonicus*) analyzed here [9], which is not in agreement with the results of the present study. This fact is probably due to the wide variation in FA profiles found in fishes depending on the catch season.

Although by-products are not edible parts of the fish, they have potential as low-cost sources of EPA and DHA, especially if they are high in lipid content and high in these n-3 PUFAs. The anchovy by-products analyzed in this work showed lower levels of EPA and DHA than those found in the fillet, but still their proportion of both PUFAs is considerable, especially in the bones (22.5–23.0% of EPA + DHA). The levels of total SFAs, MUFAs and PUFAs were similarly distributed in the head and bones, but higher values of SFA and lower values of PUFA were found in the viscera, which also showed the lowest values of EPA and DHA, making this tissue the least desirable alternative as a source of EPA and DHA, despite its considerably higher lipid content. Comparable data regarding the FA profile of anchovy (*Engraulis encrasicolus*) by-products were obtained by other authors in fishes caught in the Black Sea in Turkey, showing a higher level of SFAs (46.8%) than PUFAs (31.9%) and MUFAs (21.3%) in the viscera [21].

As in anchovy, the viscera of Chilean jack mackerel showed significantly lower levels of EPA, DHA and total PUFAs and significantly higher levels of SFAs than the rest of the analyzed by-products of this species, whereas no significantly different levels of EPA, DHA, total PUFAs and SFAs were found in the head and bones. In fact, the levels of EPA + DHA in the head and bones were close to those in the fillet. Due to their high content of EPA + DHA (23.0–23.6% in the head and 23.9–25.0% in the bone), both by-products have potential as a source for the production of n-3 PUFA oils and concentrates, as their content of SFAs such as palmitic and myristic acids could be greatly reduced by technological approaches such as low temperature crystallization [41].

Among the by-products of chub mackerel analyzed in this work, the bones showed significantly higher EPA and DHA levels and a lower SFA level than the head and viscera and close PUFA and SFA levels to those found in the fillet. Thus, the bone of chub mackerel is emerging as an interesting potential source of oils and concentrates enriched in n-3 PUFAs with lower SFA levels compared to the head and viscera. Cho et al. reported 39.2% SFAs, 27.2% MUFAs and 33.6% PUFAs (of which were 7.2% EPA and 23.0% DHA) in the bones of chub mackerel (*Scomber japonicus*) collected in a fishing company in Korea [42], which are closer values to the ones found in the present study except for the DHA level. Other authors found levels of 31% SFAs, 22% MUFAs and 40% PUFAs (of which were 9% EPA and 22% DHA) in the head of blue mackerel (*Scomber australasicus*) provided by a commercial fish processing plant in New Zealand [11] and 30% SFAs, 28% MUFAs and 24% n-3 PUFAs (of which were 3.2% EPA and 15.6% DHA) in the liver of blue mackerel caught in the China Sea, although such values were highly dependent on the fishing season [31].

Together with EPA and DHA, OA is a relevant unsaturated FA for the prevention of adverse cardiovascular events and the management of the metabolic syndrome and obesity [43,44]. Although OA is generally supplied in the diet by vegetable oils such as those from olive, rapeseed, sesame, walnut, avocado, etc. [45], fish lipids can also be a rich source of this FA [19,31,36,40], as was found in this study, particularly in the edible and non-edible parts of Chilean jack mackerel and chub mackerel.

Since fish by-products are not directly intended for human consumption, it is necessary to extract the lipids they contain, and although the Folch method is suitable for analytical purposes, more environmentally friendly and safer extraction protocols must be applied to avoid the use of chlorinated solvents. The use of the mix of *n*-hexane and 2-propanol was developed by Hara and Radin [29] and offers a simpler and more affordable alternative that has been already assayed in previous works to extract PUFA-rich lipids [30,46]. In the present study, both EPA and DHA levels were not significantly altered in the lipid extracted from most fish by-products when *n*-hexane and 2-propanol were used instead of the Folch extraction, thus indicating that such a solvent combination is a suitable alternative for lipid extraction.

In addition to FA profiles, there are several indices to assess lipid quality from a nutritional and health perspective. For products of marine origin, some of the most commonly used indices are the n-6/n-3 PUFA ratio, the atherogenic index (AI), the thrombogenic index (TI), the hypocholesterolemic/hypercholesterolemic fatty acid ratio (HH) and the fish lipid quality index (FLQ).

Given that current Western dietary patterns result in a much higher consumption of n-6 PUFA than n-3 PUFA and that n-6 PUFA are precursors of pro-inflammatory mediators, whereas n-3 PUFA are precursors of anti-inflammatory mediators, one of the most important dietary recommendations to reduce the prevalence of inflammatory diseases is to increase the consumption of n-3 PUFA-rich foods [47,48]. Consequently, low n-6/n-3 PUFA ratios are desirable to balance the intake of both types of PUFA. In this work, the lowest n-6/n-3 PUFA ratios (<0.2) were found in fillets and bones of the three fish species analyzed, as well as in the head of Chilean jack mackerel, suggesting the health-promoting potential of these products. However, all the tissues analyzed in this study showed n-6/n-3 PUFA ratios lower than 0.4.

Regarding AI, values lower than 1.5 are generally considered acceptable [49]. The lower the AI value, the better the nutritional quality of the lipid fraction, since the proportion of unsaturated FAs (considered anti-atherogenic) is higher than that of SFAs (considered pro-atherogenic). Most of the AI values found in the present study are below 1.5, although the edible part of the three fish species analyzed (fillets) showed the lowest values, together with the head and bones of Chilean jack mackerel and the bones of chub mackerel. The TI estimated the thrombogenic capacity of the lipids, although more weight is given to n-3 PUFA compared to the AI calculation, as they are more widely recognized as promoters of cardiovascular health, values lower than 1.15 being considered beneficial [49]. Most TI values found in this work are below 0.50, which characterized them as products with low thrombogenic capacity according to this index. Only the viscera of anchovy, Chilean jack mackerel and chub mackerel and the head of chub mackerel showed higher values (0.53–1.30).

The HH index serves as a more precise tool for evaluating how the fatty acid composition may influence the risk of cardiovascular disease, surpassing the PUFA/SFA ratio in accuracy. In this context, a higher HH index value indicates a greater overall protective effect against cardiovascular issues [50]. The highest HH values (>1.75) were found in the fillets of the three analyzed species, as well as in the head of the Chilean jack mackerel and the bones of the Chilean jack mackerel and the chub mackerel, indicating that these tissues contain health-promoting lipids. HH values for fillets of chub mackerel and Chilean jack mackerel are similar to those found for the same tissues and fish species in one of our previous studies [9]. Usually, HH values between 1.5 and 3.0 are found in fish tissues [51].

The FLQ index indicates the correlation between EPA and DHA and the total fatty acids. In raw fish, FLQ values typically range from 13 to 36 [50]. A higher FLQ value is preferred for nutritional reasons, as it indicates higher levels of EPA and/or DHA. The fillet of anchovy showed the highest FLQ values, as well as all anchovy by-products and the head of Chilean jack mackerel and the bones of chub mackerel and Chilean jack mackerel.

In Chile, anchovies are not intended for human consumption, and fish by-products are either discarded or used to produce fishmeal. However, the data obtained in this study on FLQ and other lipid quality indices suggest that anchovy fillets may be suitable for human consumption as a source of marine n-3 PUFA, as is the case in other countries, and that certain fish processing by-products are interesting sources for the production of EPA- and DHA-rich lipids with nutritional or nutraceutical uses, thus contributing to the valorization of these materials in line with the Sustainable Development Goals of the 2030 Agenda [52].

### 3.3. α-Tocopherol, Squalene and Cholesterol Content

Of all the vitamin E isoforms, α-tocopherol is the most abundant in fish tissues and also the most bioavailable owing to its strong affinity for the hepatic α-T transfer protein (α-TPP) [53]. The current recommended dietary allowance of α-tocopherol is 15 mg per day for adults according to the US National Institute of Health and the US Institute of Medicine [54].

Previous studies have reported the amount of α-tocopherol in fillets of the mackerel species. For example, values of 0.47 and 0.45 mg/100 g were found in the flesh of Indian mackerel (*Rastrelliger kanagurta*) from Turkey and Atlantic mackerel (*Scomber scombrus*) from Tunisia, respectively [55,56]. Other authors observed higher values of α-tocopherol in fillets of Atlantic mackerel (*Scomber scombrus*) from Canada (1.3 mg/100 g) and Turkey (1.0 mg/100 g) [57,58]. However, to the best of our knowledge, the amount of α-tocopherol available in the chub mackerel (*Scomber japonicus*) has not been reported yet. In this work, 0.57 mg/100 g of α-tocopherol was found in the fillet of chub mackerel, which is within the expected range as shown above for other species from the *Scomber* genus. Similar levels of α-tocopherol have been found in horse mackerel (*Trachurus trachurus*) fillets, ranging from 0.45 to 0.64 mg/100 g, depending on the fishing season [39]. These data are similar to the amount found in the present work in Chilean jack mackerel (*Trachurus murphyi*) fillets. Regarding anchovy, information on vitamin E content is scarce. Polat et al. found 4.1 mg of α-tocopherol in 100 g anchovy (*Engraulis encrasicolus*) muscle [59].

The richest food sources of squalene are shark liver oil, as well as several vegetable oils such as those of olive and palm [15]. In comparison, the amounts of squalene found in fillets of the analyzed fish species in this work are much lower. These results agree with a previous study that found squalene amounts up to 1.83 mg/100 g in salted-dried fishes, most of them with lower levels than 0.4 mg/100 g [60]. Similarly, other authors reported amounts up to 1.0 mg/100 g in raw fillet of horse mackerel (*Trachurus trachurus*) [39].

Ozogul et al. reported cholesterol levels in the muscle of several fish species, including chub mackerel (*Scomber japonicus*) with 38.9 mg/100 g, which is similar to that found in the present work [61]. Other species studied by these authors, belonging to the same genus as those analyzed in the present work, were mackerel (*Scomber scombrus*) (32.1 mg cholesterol/100 g), Atlantic horse mackerel (*Trachurus trachurus*) (42.5 mg/100 g), Mediterranean horse mackerel (*Trachurus mediterraneus*) (32.5 mg/100 g) and anchovy (*Engraulis encrasicolus*) (57.3 mg/100 g) [61]. These cholesterol amounts are generally lower than the levels found in meat and poultry such as beef, pork, chicken or lamb [62].

Although some data on the amount of tocopherol, squalene and cholesterol in fish fillets have been reported in the literature, little is known about the levels of these compounds in fish by-products. However, given their potential as raw materials for the production of n-3 PUFA-rich oils and the fact that tocopherol, squalene and cholesterol are lipophilic compounds that are easily solubilized in the oils, having information on this topic is a relevant issue. The present study includes new data on α-tocopherol content in by-products (viscera, bones and head) of anchovy, chub mackerel and Chilean jack mackerel. Among all the analyzed by-products, the highest amounts of α-tocopherol were found in the bones and head of chub mackerel. Considering the amount of water and lipid content in these by-products, 100 g of crude oil extracted from the bones and head of chub mackerel would contain 16.1 and 15.5 mg of α-tocopherol, respectively. Regarding squalene, the viscera of chub mackerel was the by-product that showed the highest content, but it was still far from the amounts of squalene found in some commonly consumed vegetable foods such as olive or palm oil. In terms of cholesterol, almost all analyzed fish by-products had higher levels than fillets, and therefore caution should be exercised whether using these by-products to produce n-3 PUFA oils with nutritional or nutraceutical purposes. Technological approaches such as vacuum stripping or the use of beta-cyclodextrins could be considered to remove cholesterol from the extracted oils [30].

## 4. Materials and Methods

### 4.1. Reagents and Samples

Unless otherwise stated, all solvents and reagents used in this work were from Merck (Darmstadt, Germany).

Whole fresh specimens of Chilean jack mackerel (*Trachurus murphyi*) (*n* = 8), chub mackerel (*Scomber japonicus*) (*n* = 8) and anchovy (*Engraulis ringens*) (*n* = 100) were provided by local fishermen in the Coquimbo port (Coquimbo, Chile) in March–April, May–June, and August–September 2023, respectively. The selection of these dates ensures that the samples are representative of the species available during the months of greatest fishing activity. All fishes were immediately frozen at −20 °C and delivered to the laboratory, where they were thawed and eviscerated. Four parts of each specimen (fillet, head, viscera and bones) were separated, homogenized in a food processor (Ursus Trotter, model UT-METALLER605P 1.5 L), frozen at −80 °C and then lyophilized for 48 h (MFD-1050M model lyophilizer from MLAB) to remove water. The moisture content of each sample was determined by the difference in weight before and after lyophilization, and the samples were stored at −20 °C in sealed polyethylene bags under a nitrogen atmosphere until further processing.

### 4.2. Lipid Extraction

Two methods were used for lipid extraction from fish tissues: (i) the Folch method was carried out by adding 20 mL of chloroform:methanol (2:1 *v*/*v*) to 1 g of lyophilized sample and magnetically stirring the mixture for 1 h at room temperature in the darkness under a nitrogen inert atmosphere in a sealed glass flask [63]. The content of the flask was then filtered, and the filtrate was placed in a glass separatory funnel with 5 mL of an aqueous solution of magnesium chloride (9% *w*/*v*). After vigorous shaking for 2 min, the resulting lower organic phase was collected and filtered through anhydrous sodium sulfate, and then the solvent was removed in a rotary evaporator at 40 °C. The residual oil was collected, weighed and stored at −20 °C under a nitrogen atmosphere to prevent lipid degradation until further use; (ii) the extraction with *n*-hexane:2-propanol was carried out according to Rincón-Cervera et al. with some modifications [30]. The lyophilized samples (1 g) were mixed with 10 mL of *n*-hexane:2-propanol (3:2 *v*/*v*) under a nitrogen atmosphere in a sealed glass flask and magnetically stirred for 30 min at room temperature in the darkness under an inert nitrogen atmosphere. Then, the mixture was filtrated and the solid residue was extracted again with a fresh portion of solvent (10 mL) for 30 min. Both filtrates were put together, and the solvent was removed in a rotary evaporator at 40 °C. The resulting lipids were collected, weighed and stored at −20 °C in the dark under a nitrogen atmosphere until further use.

### 4.3. Fatty Acid Analysis

The FA profiles of the extracted fish lipids were obtained after derivatization to FA methyl esters (FAME), as described in a previous work [8]. Briefly, 50 mg of lipids were weighed into test tubes, and then 1 mL of *n*-hexane was added to each one. FAME were obtained by adding 2 mL of the methylation mixture (methanol:acetyl chloride 20:1 *v*/*v*) and heating the closed tubes at 100 °C for 40 min in a hot block. After cooling at room temperature, 1 mL of distilled water was added to each tube, followed by centrifugation at 3500 rpm for 3 min. The upper hexane layer containing FAME was collected for analysis by gas chromatography coupled with flame ionization detection (GC-FID) (Agilent 6890N with a 7683B autosampler, Agilent Technologies, Santa Clara, CA, USA). The initial oven temperature was set at 140 °C and kept constant for 5 min, then increased at 4 °C/min to 190 °C, followed at 1 °C/min to 220 °C and then at 4 °C/min to 240 °C, the temperature being kept at 240 °C for 5 min. Nitrogen was used as a carrier gas and the split ratio was set at 1:100. The temperatures of the injector and detector were set at 270 and 260 °C, respectively. A Supelco SP-2560 capillary column (100 m × 0.25 mm × 0.2 μm film) was used to carry out the analysis (Sigma-Aldrich, Madrid, Spain). FAME were identified according to their respective retention times compared to a known analytical standard (37 component FAME mix from Supelco, Sigma-Aldrich). The peaks corresponding to eicosatetraenoic acid (C20:4 n-3) and docosapentaenoic acid (DPA, C22:5 n-3) were identified based on the literature [11,64].

### 4.4. Lipid Nutritional Quality Indices

Four indices were calculated for each fish species based on FA composition [9]. Atherogenic index (AI), thrombogenic index (TI), hypocholesterolemic/hypercholesterolemic FA ratio (HH) and fish lipid quality index (FLQ) were calculated as follows:

AI = [(4 × 14:0) + 16:0]/Σ Unsaturated FA.(1)

TI = (14:0 + 16:0 + 18:0)/[(0.5 × Σ MUFA) + (0.5 × Σ n-6 PUFA) + (3 × Σ n-3 PUFA) + (Σ n-3 PUFA/Σ n-6 PUFA)].(2)

HH = [(18:1 n-9 + 18:1 n-7) + Σ PUFA]/(14:0 + 16:0).(3)

FLQ = 100 × (20:5 n-3 + 22:6 n-3)/ΣFA.(4)

### 4.5. Analysis of α-Tocopherol

The amount of α-tocopherol was quantified in the fish lipids extracted with the Folch method by dissolving 250 mg of oil in 1 mL of HPLC-grade *n*-hexane and injecting 20 µL of this solution into HPLC equipment (Agilent 1260 Technologies, Santa Clara, CA, USA), equipped with a Spherisorb Silica-80 column (particle size 5 μm, dimensions 4.6 mm × 250 mm, 5 μm particle size) (Waters, MA, USA) and a UV-Vis detector coupled to a computer running ChemStation software B.04.03 version [65]. The mobile phase consisted of *n*-hexane:2-propanol (99:1 *v*/*v*) at a flow rate of 1 mL/min. Detection was carried out at a wavelength of 292 nm. An analytical standard of α-tocopherol (purity ≥ 95%) from Cayman Chemical (Ann Arbor, MI, USA) was used for identification and quantification purposes.

### 4.6. Analysis of Squalene and Cholesterol

Squalene and cholesterol were analyzed in the fish lipids extracted with the Folch method according to the method described by Spiric et al. with some modifications [66]. Briefly, an aliquot of fish lipids (500 mg) was mixed with 10 mL of a KOH solution in methanol (0.5 M) in a sealed glass flask and vortexed for 30 s. The mixture was saponified at 80 °C for 1 h, and then it was cooled at room temperature, and 10 mL of distilled water was added. The mixture was placed in a separatory funnel, and two phases were formed after the addition of 15 mL diethyl ether:*n*-hexane (1:1 *v*/*v*). The upper organic phase was collected, and the hydroalcoholic phase was extracted twice more with fresh volumes of diethyl ether:*n*-hexane. After combining the three organic phases, the solvent was removed in a rotary evaporator and the residue was dissolved in 500 μL of acetonitrile:2-propanol (65:35 *v*/*v*) for HPLC analysis. An aliquot (20 μL) of the solution was injected into an HPLC system (Agilent 1260 Technologies, Santa Clara, CA, USA), equipped with a ProntoSIL KromaPlus C18 column (250 × 4.6 mm, 5 μm particle size) (Bischoff, Leonberg, Germany) and a UV-Vis detector coupled to a computer running ChemStation software. The mobile phase consisted of acetonitrile:2-propanol (65:35 *v*/*v*) with an isocratic flow rate of 1 mL/min. Detection was carried out at a wavelength of 210 nm. Analytical standards of squalene and cholesterol from Cayman Chem (Ann Arbor, MI, USA) were used for identification and quantification.

### 4.7. Statistical Analysis

All the analyses were carried out in triplicate and the results are reported as mean ± standard deviation (SD). The comparison between groups was made to yield FA profile, α-tocopherol, cholesterol and squalene content using one way and two way ANOVA parametric test; after verification of the normality of distributions and homogeneity of variances in the data, *p*-value < 0.05 was considered significant. A post hoc analysis using the Tukey test for intergroup differences comparison was conducted. JMPStata, version 14 and Graph Pad Prism 8 software were used for the statistical analysis.

## 5. Conclusions

The valorization of fish by-products as raw materials to obtain n-3 PUFA-containing oils contributes to the reduction of food waste in line with the Sustainable Development Goals of the United Nations 2030 Agenda, promotes more sustainable practices within the food chain, minimizes environmental impacts and responds to the growing demand for healthy marine ingredients and nutraceuticals. However, information on the FA composition and bioactive compounds present in fish by-products is scarce in the literature.

Chile has a long coastline, and the fishing sector is one of the most important economic activities in the country. The generation of by-products from fish processing is a major problem, with anchovy (*E. ringens*), Chilean jack mackerel (*T. murphyi*) and chub mackerel (*S. japonicus*) accounting for a high percentage of the by-products generated. This work not only provides valuable nutritional information on the lipid content, FA profile, lipid quality indices and amounts of α-tocopherol, squalene and cholesterol in the fillets of the above-mentioned fish species but also provides novel data on the characterization of their main by-products (head, bones and viscera), which may be interesting inputs for the nutraceutical industry dedicated to the production of marine oils, n-3 PUFA concentrates and dietary supplements. The information provided by this cross-sectional study should be complemented by further research considering the potential effects of seasonal, environmental and biological factors on the composition of the lipid fraction of wild fishes caught along the Chilean coast.

## Figures and Tables

**Table 1:** Moisture (%) and lipid amount (g/100 g dry weight) of fillets and by-products of anchovy, Chilean jack mackerel and chub mackerel. The lipid fraction was extracted by the Folch method and using a mixture of *n*-hexane and 2-propanol (Hex:2-PrOH).

	Anchovy				Chilean Jack Mackerel				Chub Mackerel			
	Viscera	Bones	Head	Fillet	Viscera	Bones	Head	Fillet	Viscera	Bones	Head	Fillet
Moisture (%)	72.6 ± 0.8 ^D^	69.5 ± 0.6 ^C^	72.3 ± 0.8 ^D^	76.0 ± 0.8 ^E^	70.7 ± 1.1 ^CD^	64.6 ± 0.7 ^B^	69.8 ± 1.0 ^C^	72.3 ± 0.4 ^D^	66.1 ± 0.6 ^B^	65.6 ± 0.8 ^B^	69.7 ± 0.5 ^C^	62.0 ± 0.6 ^A^
Lipid amount (g/100 g dw)												
Folch	25.6 ± 1.5 ^aA^	14.5 ± 1.2 ^aC^	17.0 ± 0.5 ^aB^	9.6 ± 0.9 ^aD^	21.8 ± 2.0 ^aA^	11.8 ± 0.8 ^aB^	20.5 ± 0.7 ^aA^	10.1 ± 0.9 ^aC^	45.8 ± 1.5 ^aA^	24.4 ± 1.3 ^aB^	25.1 ± 0.4 ^aB^	26.4 ± 2.5 ^aB^
Hex:2-PrOH	22.3 ± 1.9 ^aA^	13.9 ± 0.4 ^aB^	12.5 ± 1.0 ^bB^	5.4 ± 0.5 ^bC^	18.7 ± 0.8 ^bB^	12.6 ± 0.4 ^aC^	20.5 ± 1.1 ^aA^	10.6 ± 0.8 ^aD^	45.6 ± 0.9 ^aA^	27.0 ± 1.5 ^aB^	20.4 ± 1.1 ^bC^	29.2 ± 1.8 ^aB^

Results are reported as mean value ± standard deviation (*n* = 3). Different superscript lowercase letters in each column mean significantly different values (*p* < 0.05) between extraction methods for each tissue, and different superscript uppercase letters in each row for each fish species mean significantly different values among tissues (*p* < 0.05). Moisture percentages were compared among all fish species and tissues, and different superscript uppercase letters in the row means significantly different values (*p* < 0.05).

**Table 2:** Fatty acid profile (% of total FAs) and nutritional indices of lipids extracted from anchovy fillets and by-products.

Fatty Acids	Fillet		Head		Bones		Viscera	
	Folch	Hex:2-PrOH	Folch	Hex:2-PrOH	Folch	Hex:2-PrOH	Folch	Hex:2-PrOH
C14:0	6.91 ± 0.02 ^a^	7.50 ± 0.23 ^a^	10.75 ± 0.12 ^b^	10.61 ± 0.25 ^b^	10.81 ± 0.21 ^bc^	11.42 ± 0.42 ^c^	12.33 ± 0.20 ^d^	12.99 ± 0.19 ^e^
C15:0	0.33 ± 0.02 ^a^	0.39 ± 0.02 ^a^	0.57 ± 0.01 ^bc^	0.60 ± 0.05 ^c^	0.46 ± 0.03 ^ab^	0.57 ± 0.09 ^bc^	0.53 ± 0.05 ^bc^	0.62 ± 0.07 ^c^
C16:0	21.54 ± 0.21 ^a^	20.82 ± 0.13 ^a^	25.82 ± 0.47 ^c^	25.02 ± 0.55 ^c^	23.65 ± 0.18 ^b^	23.66 ± 0.35 ^b^	27.19 ± 0.49 ^d^	28.92 ± 0.31 ^e^
C17:0	0.39 ± 0.03 ^ab^	0.37 ± 0.02 ^a^	0.57 ± 0.04 ^cd^	0.54 ± 0.03 ^bcd^	0.44 ± 0.02 ^abc^	0.48 ± 0.09 ^abc^	0.65 ± 0.09 ^d^	0.68 ± 0.07 ^d^
C18:0	3.93 ± 0.02 ^ab^	3.51 ± 0.10 ^a^	5.68 ± 0.34 ^e^	4.86 ± 0.11 ^d^	4.37 ± 0.07 ^c^	4.30 ± 0.11 ^bc^	5.88 ± 0.13 ^ef^	6.17 ± 0.10 ^f^
C20:0	0.07 ± 0.01 ^a^	0.09 ± 0.00 ^a^	0.20 ± 0.02 ^c^	0.17 ± 0.01 ^bc^	0.15 ± 0.02 ^b^	0.16 ± 0.03 ^bc^	0.15 ± 0.01 ^b^	0.15 ± 0.02 ^b^
C24:0	0.06 ± 0.01 ^ab^	0.09 ± 0.01 ^bc^	0.12 ± 0.03 ^c^	0.08 ± 0.00 ^bc^	0.09 ± 0.02 ^bc^	0.06 ± 0.01 ^ab^	0.03 ± 0.01 ^a^	0.09 ± 0.01 ^bc^
Σ SFA	33.24 ± 0.22 ^a^	32.77 ± 0.29 ^a^	43.71 ± 0.59 ^c^	41.87 ± 0.62 ^bc^	39.97 ± 0.29 ^b^	40.66 ± 0.57 ^b^	46.76 ± 0.55 ^d^	49.62 ± 0.39 ^e^
C16:1 n-7	7.12 ± 0.03 ^a^	8.79 ± 0.50 ^b^	11.83 ± 0.31 ^c^	11.74 ± 0.18 ^c^	12.05 ± 0.07 ^c^	11.82 ± 0.67 ^c^	11.64 ± 0.14 ^c^	12.42 ± 0.10 ^c^
C18:1 n-9	7.12 ± 0.02 ^a^	8.37 ± 0.25 ^b^	11.25 ± 0.06 ^f^	10.71 ± 0.21 ^de^	10.82 ± 0.16 ^def^	11.01 ± 0.37 ^ef^	9.97 ± 0.07 ^c^	10.30 ± 0.02 ^cd^
C18:1 n-7	3.57 ± 0.01 ^b^	3.24 ± 0.08 ^a^	4.06 ± 0.19 ^c^	3.69 ± 0.10 ^b^	3.70 ± 0.04 ^b^	3.67 ± 0.12 ^b^	4.14 ± 0.12 ^c^	4.24 ± 0.05 ^c^
C20:1 n-9	0.22 ± 0.01 ^a^	0.28 ± 0.04 ^ab^	0.38 ± 0.01 ^ab^	0.48 ± 0.23 ^b^	0.34 ± 0.02 ^ab^	0.42 ± 0.06 ^ab^	0.35 ± 0.02 ^ab^	0.39 ± 0.02 ^ab^
C24:1 n-9	0.37 ± 0.04 ^a^	0.42 ± 0.07 ^a^	1.35 ± 0.03 ^e^	1.10 ± 0.15 ^de^	0.94 ± 0.15 ^cd^	0.99 ± 0.13 ^cd^	0.63 ± 0.06 ^ab^	0.75 ± 0.11 ^bc^
Σ MUFA	18.39 ± 0.05 ^a^	21.10 ± 0.57 ^b^	28.86 ± 0.38 ^d^	27.72 ± 0.40 ^cd^	27.85 ± 0.23 ^cd^	27.91 ± 0.78 ^cd^	26.73 ± 0.21 ^c^	28.10 ± 0.16 ^d^
C18:2 n-6	2.10 ± 0.05 ^a^	2.17 ± 0.49 ^a^	4.25 ± 0.23 ^cd^	4.94 ± 0.09 ^d^	3.47 ± 0.64 ^bc^	2.83 ± 0.15 ^ab^	4.14 ± 0.48 ^cd^	4.27 ± 0.19 ^cd^
C20:2 n-6	0.21 ± 0.03 ^a^	0.25 ± 0.04 ^a^	0.23 ± 0.04 ^a^	0.21 ± 0.01 ^a^	0.26 ± 0.02 ^a^	0.25 ± 0.02 ^a^	0.22 ± 0.03 ^a^	0.22 ± 0.03 ^a^
C20:4 n-6	0.90 ± 0.08 ^b^	0.90 ± 0.10 ^b^	0.56 ± 0.11 ^a^	0.74 ± 0.04 ^ab^	0.75 ± 0.12 ^ab^	0.70 ± 0.09 ^ab^	0.52 ± 0.03 ^a^	0.57 ± 0.10 ^a^
C18:3 n-3	0.50 ± 0.02 ^a^	0.59 ± 0.01 ^ab^	0.64 ± 0.02 ^bc^	0.74 ± 0.00 ^c^	0.71 ± 0.04 ^c^	0.74 ± 0.09 ^c^	0.56 ± 0.05 ^ab^	0.63 ± 0.02 ^bc^
C18:4 n-3	1.57 ± 0.04 ^ab^	2.36 ± 0.16 ^c^	1.85 ± 0.12 ^b^	2.39 ± 0.11 ^c^	2.31 ± 0.09 ^c^	2.50 ± 0.15 ^c^	1.69 ± 0.11 ^ab^	1.39 ± 0.16 ^a^
C20:4 n-3	0.47 ± 0.08 ^abc^	0.60 ± 0.04 ^c^	0.45 ± 0.03 ^ab^	0.50 ± 0.05 ^abc^	0.49 ± 0.05 ^abc^	0.55 ± 0.05 ^bc^	0.55 ± 0.06 ^bc^	0.38 ± 0.02 ^a^
C20:5 n-3 (EPA)	18.88 ± 0.13 ^d^	20.56 ± 0.33 ^e^	10.64 ± 0.54 ^b^	11.87 ± 0.59 ^b^	14.73 ± 0.38 ^c^	14.70 ± 0.32 ^c^	11.29 ± 0.77 ^b^	9.07 ± 0.60 ^a^
C22:5 n-3	1.70 ± 0.02 ^d^	1.82 ± 0.11 ^d^	0.95 ± 0.15 ^ab^	1.09 ± 0.13 ^bc^	1.22 ± 0.04 ^c^	1.33 ± 0.10 ^c^	1.23 ± 0.03 ^c^	0.82 ± 0.04 ^a^
C22:6 n-3 (DHA)	22.05 ± 0.23 ^e^	16.87 ± 0.51 ^d^	7.87 ± 0.49 ^c^	7.90 ± 0.46 ^c^	8.24 ± 0.16 ^c^	7.82 ± 0.16 ^c^	6.30 ± 0.62 ^b^	4.92 ± 0.46 ^a^
Σ PUFA	48.37 ± 0.29 ^d^	46.12 ± 0.81 ^d^	27.43 ± 0.80 ^b^	30.39 ± 0.77 ^c^	32.19 ± 0.78 ^c^	31.43 ± 0.44 ^c^	26.50 ± 1.11 ^b^	22.28 ± 0.80 ^a^
Lipid quality indices								
n-6/n-3 PUFA	0.07	0.08	0.23	0.24	0.16	0.14	0.23	0.29
AI	0.74	0.76	1.22	1.16	1.11	1.17	1.44	1.61
TI	0.20	0.21	0.48	0.43	0.37	0.37	0.53	0.67
HH	2.08	2.04	1.17	1.26	1.35	1.31	1.03	0.88
FLQ	40.93	37.43	18.50	19.77	22.97	22.53	17.60	14.00

Results are reported as mean ± standard deviation (*n* = 3). Different superscript letters in each row mean significantly different values (*p* < 0.05). SFA: saturated FA; MUFA: monounsaturated FA; PUFA: polyunsaturated FA; AI: atherogenic index; TI: thrombogenic index; HH: hypocholesterolemic/hypercholesterolemic index; FLQ: fish lipid quality index.

**Table 3:** Fatty acid profile (% of total FAs) and nutritional indices of lipids extracted from Chilean jack mackerel fillets and by-products.

Fatty Acids	Fillet		Head		Bones		Viscera	
	Folch	Hex:2-PrOH	Folch	Hex:2-PrOH	Folch	Hex:2-PrOH	Folch	Hex:2-PrOH
C14:0	4.55 ± 0.74 ^ab^	4.36 ± 0.63 ^a^	5.87 ± 0.37 ^bcd^	6.32 ± 0.02 ^cd^	5.33 ± 0.84 ^abc^	5.69 ± 0.30 ^abcd^	6.87 ± 0.32 ^d^	4.77 ± 0.12 ^ab^
C15:0	0.30 ± 0.05 ^a^	0.39 ± 0.04 ^a^	0.33 ± 0.17 ^a^	0.40 ± 0.20 ^ab^	0.36 ± 0.04 ^a^	0.41 ± 0.07 ^ab^	0.67 ± 0.03 ^b^	0.42 ± 0.03 ^ab^
C16:0	26.16 ± 1.50 ^b^	24.27 ± 0.62 ^ab^	23.85 ± 0.93 ^a^	23.05 ± 0.52 ^a^	24.97 ± 0.14 ^ab^	23.86 ± 0.66 ^a^	35.20 ± 0.73 ^c^	33.60 ± 0.38 ^c^
C17:0	0.28 ± 0.02 ^a^	0.60 ± 0.13 ^ab^	0.33 ± 0.18 ^a^	0.45 ± 0.02 ^ab^	0.42 ± 0.27 ^ab^	0.35 ± 0.02 ^a^	0.70 ± 0.05 ^b^	0.55 ± 0.04 ^ab^
C18:0	6.39 ± 0.26 ^b^	5.91 ± 0.52 ^ab^	5.37 ± 0.10 ^a^	5.39 ± 0.13 ^a^	5.62 ± 0.13 ^a^	5.86 ± 0.16 ^ab^	7.87 ± 0.31 ^c^	10.12 ± 0.13 ^d^
C20:0	0.16 ± 0.01 ^ab^	0.16 ± 0.02 ^ab^	0.08 ± 0.01 ^a^	0.13 ± 0.05 ^ab^	0.13 ± 0.03 ^ab^	0.14 ± 0.02 ^ab^	0.22 ± 0.08 ^b^	0.12 ± 0.02 ^ab^
C24:0	n.d.	n.d.	n.d.	n.d.	n.d.	0.10 ± 0.04	n.d.	n.d.
ΣSFA	37.83 ± 1.69 ^a^	35.68 ± 1.03 ^a^	35.83 ± 1.03 ^a^	35.74 ± 0.58 ^a^	36.83 ± 0.91 ^a^	36.42 ± 0.75 ^a^	51.54 ± 0.86 ^b^	49.58 ± 0.42 ^b^
C16:1 n-7	3.74 ± 0.83 ^a^	5.15 ± 0.45 ^b^	6.85 ± 0.58 ^cde^	7.32 ± 0.41 ^de^	5.69 ± 0.05 ^bc^	6.36 ± 0.57 ^bcd^	7.94 ± 0.18 ^e^	8.02 ± 0.02 ^e^
C18:1 n-9	22.07 ± 0.80 ^bc^	19.00 ± 1.03 ^a^	20.08 ± 1.04 ^ab^	19.19 ± 0.51 ^a^	19.63 ± 0.38 ^a^	18.72 ± 0.80 ^a^	22.13 ± 0.21 ^c^	30.34 ± 0.56 ^d^
C18:1 n-7	3.11 ± 0.44 ^a^	3.29 ± 0.25 ^a^	3.80 ± 0.41 ^ab^	4.13 ± 0.15 ^b^	3.06 ± 0.04 ^a^	3.77 ± 0.26 ^ab^	5.22 ± 0.38 ^c^	5.28 ± 0.10 ^c^
C20:1 n-9	0.63 ± 0.13 ^a^	0.91 ± 0.18 ^ab^	1.06 ± 0.29 ^ab^	1.20 ± 0.25 ^ab^	0.70 ± 0.02 ^ab^	0.91 ± 0.36 ^ab^	1.03 ± 0.19 ^ab^	1.29 ± 0.05 ^b^
C24:1 n-9	0.55 ± 0.12 ^a^	0.63 ± 0.12 ^a^	0.65 ± 0.10 ^a^	0.62 ± 0.06 ^a^	0.96 ± 0.05 ^b^	0.65 ± 0.04 ^a^	0.67 ± 0.07 ^a^	0.56 ± 0.12 ^a^
ΣMUFA	30.10 ± 1.24 ^a^	28.98 ± 1.17 ^a^	32.44 ± 1.29 ^b^	32.47 ± 0.72 ^b^	30.04 ± 0.39 ^a^	30.42 ± 1.08 ^a^	36.99 ± 0.51 ^c^	45.50 ± 0.58 ^d^
C18:2 n-6	1.98 ± 0.29 ^b^	1.84 ± 0.74 ^b^	2.14 ± 0.17 ^b^	2.28 ± 0.08 ^b^	2.50 ± 0.29 ^b^	2.25 ± 0.08 ^b^	1.88 ± 0.25 ^b^	0.81 ± 0.06 ^a^
C20:2 n-6	0.20 ± 0.06 ^a^	0.30 ± 0.09 ^a^	0.20 ± 0.02 ^a^	0.18 ± 0.06 ^a^	0.19 ± 0.02 ^a^	0.20 ± 0.01 ^a^	0.14 ± 0.03 ^a^	0.20 ± 0.11 ^a^
C20:4 n-6	1.14 ± 0.12 ^cd^	1.37 ± 0.05 ^cd^	1.15 ± 0.25 ^cd^	1.02 ± 0.12 ^bc^	1.45 ± 0.06 ^d^	1.21 ± 0.08 ^cd^	0.68 ± 0.23 ^b^	0.26 ± 0.03 ^a^
C18:3 n-3	0.48 ± 0.31 ^a^	0.67 ± 0.38 ^a^	0.60 ± 0.26 ^a^	0.61 ± 0.24 ^a^	0.55 ± 0.21 ^a^	0.45 ± 0.17 ^a^	0.10 ± 0.03 ^a^	0.41 ± 0.22 ^a^
C18:4 n-3	0.63 ± 0.10 ^abc^	0.78 ± 0.07 ^c^	0.81 ± 0.21 ^c^	0.85 ± 0.26 ^c^	0.82 ± 0.15 ^c^	0.74 ± 0.08 ^bc^	0.36 ± 0.06 ^ab^	0.29 ± 0.03 ^a^
C20:4 n-3	0.48 ± 0.19 ^bc^	0.60 ± 0.10 ^c^	0.52 ± 0.09 ^c^	0.49 ± 0.08 ^bc^	0.52 ± 0.10 ^c^	0.48 ± 0.05 ^bc^	0.22 ± 0.03 ^ab^	0.12 ± 0.02 ^a^
C20:5 n-3 (EPA)	8.73 ± 0.74 ^c^	10.00 ± 0.23 ^d^	11.31 ± 0.51 ^e^	11.75 ± 0.17 ^e^	10.75 ± 0.31 ^de^	11.27 ± 0.05 ^e^	3.43 ± 0.22 ^b^	1.29 ± 0.37 ^a^
C22:5 n-3	3.04 ± 0.03 ^c^	3.12 ± 0.41 ^c^	3.28 ± 0.51 ^c^	2.79 ± 0.22 ^c^	3.25 ± 0.17 ^c^	2.80 ± 0.08 ^c^	1.25 ± 0.35 ^b^	0.36 ± 0.11 ^a^
C22:6 n-3 (DHA)	15.43 ± 0.96 ^cd^	16.63 ± 1.95 ^d^	11.73 ± 0.52 ^b^	11.88 ± 0.32 ^b^	13.10 ± 0.73 ^bc^	13.80 ± 0.25 ^bc^	3.42 ± 0.45 ^a^	1.18 ± 0.17 ^a^
ΣPUFA	32.12 ± 1.31 ^cd^	35.32 ± 2.17 ^d^	31.74 ± 1.00 ^c^	31.85 ± 0.58 ^c^	33.13 ± 0.91 ^cd^	33.19 ± 0.35 ^cd^	11.48 ± 0.71 ^b^	4.92 ± 0.49 ^a^
Lipid quality indices								
n-6/n-3 PUFA	0.12	0.11	0.12	0.12	0.14	0.12	0.31	0.35
AI	0.71	0.65	0.74	0.75	0.73	0.73	1.29	1.04
TI	0.33	0.29	0.32	0.31	0.32	0.31	1.01	1.30
HH	1.87	2.01	1.87	1.88	1.84	1.88	0.92	1.06
FLQ	24.16	26.63	23.04	23.63	23.85	25.06	6.84	2.47

Results are reported as mean ± standard deviation (*n* = 3). Different superscript letters in each row mean significantly different values (*p* < 0.05). SFA: saturated FA; MUFA: monounsaturated FA; PUFA: polyunsaturated FA; AI: atherogenic index; TI: thrombogenic index; HH: hypocholesterolemic/hypercholesterolemic index; FLQ: fish lipid quality index; n.d.: not detected.

**Table 4:** Fatty acid profile (% of total FAs) and nutritional indices of lipids extracted from chub mackerel fillets and by-products.

Fatty Acids	Fillet		Head		Bones		Viscera	
	Folch	Hex:2-PrOH	Folch	Hex:2-PrOH	Folch	Hex:2-PrOH	Folch	Hex:2-PrOH
C14:0	4.42 ± 0.11 ^ab^	4.25 ± 0.24 ^a^	6.64 ± 0.07 ^e^	7.07 ± 0.07 ^f^	4.70 ± 0.23 ^bc^	4.90 ± 0.22 ^c^	5.58 ± 0.01 ^d^	5.70 ± 0.07 ^d^
C15:0	0.45 ± 0.04 ^a^	0.48 ± 0.03 ^ab^	1.05 ± 0.23 ^c^	1.00 ± 0.03 ^c^	0.65 ± 0.03 ^ab^	0.54 ± 0.03 ^ab^	0.68 ± 0.02 ^ab^	0.70 ± 0.02 ^b^
C16:0	24.51 ± 0.42 ^ab^	24.96 ± 0.29 ^b^	30.49 ± 0.30 ^c^	30.44 ± 0.18 ^c^	23.94 ± 0.18 ^a^	23.81 ± 0.31 ^a^	30.31 ± 0.09 ^c^	30.20 ± 0.31 ^c^
C17:0	0.79 ± 0.06 ^ab^	0.76 ± 0.06 ^a^	1.41 ± 0.03 ^d^	1.53 ± 0.04 ^d^	0.85 ± 0.07 ^ab^	0.80 ± 0.06 ^ab^	1.01 ± 0.08 ^c^	0.92 ± 0.05 ^bc^
C18:0	6.17 ± 0.09 ^bc^	6.46 ± 0.14 ^c^	7.59 ± 0.06 ^d^	7.39 ± 0.07 ^d^	5.95 ± 0.24 ^b^	5.53 ± 0.12 ^a^	7.25 ± 0.09 ^d^	7.27 ± 0.03 ^d^
C20:0	0.21 ± 0.01 ^a^	0.22 ± 0.01 ^a^	0.35 ± 0.03 ^c^	0.34 ± 0.03 ^c^	0.21 ± 0.01 ^a^	0.20 ± 0.02 ^a^	0.30 ± 0.02 ^bc^	0.25 ± 0.02 ^ab^
C24:0	n.d.	n.d.	n.d.	n.d.	n.d.	n.d.	n.d.	n.d.
ΣSFA	36.55 ± 0.45 ^ab^	37.13 ± 0.41 ^b^	47.53 ± 0.39 ^d^	47.78 ± 0.21 ^d^	36.31 ± 0.38 ^ab^	35.77 ± 0.40 ^a^	45.14 ± 0.15 ^c^	45.04 ± 0.32 ^c^
C16:1 n-7	4.51 ± 0.43 ^ab^	4.24 ± 0.19 ^a^	5.69 ± 0.05 ^cd^	6.00 ± 0.06 ^d^	4.32 ± 0.17 ^ab^	4.79 ± 0.26 ^ab^	5.09 ± 0.09 ^bc^	5.65 ± 0.57 ^cd^
C18:1 n-9	21.52 ± 0.33 ^a^	21.15 ± 0.96 ^a^	23.11 ± 0.40 ^b^	23.79 ± 0.27 ^b^	20.56 ± 0.83 ^a^	21.23 ± 0.29 ^a^	26.42 ± 0.16 ^c^	26.43 ± 0.25 ^c^
C18:1 n-7	3.87 ± 0.39 ^a^	3.77 ± 0.21 ^a^	4.40 ± 0.04 ^b^	4.53 ± 0.03 ^b^	3.77 ± 0.03 ^a^	3.76 ± 0.04 ^a^	4.52 ± 0.02 ^b^	4.59 ± 0.09 ^b^
C20:1 n-9	5.87 ± 0.15 ^c^	6.10 ± 0.27 ^c^	3.74 ± 0.02 ^a^	3.84 ± 0.04 ^a^	4.83 ± 0.27 ^b^	5.06 ± 0.04 ^b^	7.80 ± 0.13 ^d^	8.01 ± 0.03 ^d^
C24:1 n-9	0.47 ± 0.04 ^a^	0.57 ± 0.06 ^ab^	1.00 ± 0.07 ^c^	1.04 ± 0.04 ^c^	0.60 ± 0.07 ^ab^	0.52 ± 0.05 ^ab^	0.67 ± 0.04 ^b^	0.62 ± 0.05 ^ab^
ΣMUFA	36.24 ± 0.68 ^bc^	35.84 ± 1.03 ^ab^	37.93 ± 0.41 ^cd^	39.21 ± 0.28 ^d^	34.08 ± 0.89 ^a^	35.37 ± 0.40 ^ab^	44.51 ± 0.22 ^e^	45.31 ± 0.63 ^e^
C18:2 n-6	1.64 ± 0.12 ^c^	1.66 ± 0.07 ^c^	1.30 ± 0.16 ^ab^	1.42 ± 0.04 ^bc^	1.64 ± 0.13 ^c^	1.56 ± 0.07 ^bc^	1.10 ± 0.05 ^a^	1.08 ± 0.03 ^a^
C20:2 n-6	0.22 ± 0.01 ^a^	0.25 ± 0.04 ^a^	0.19 ± 0.03 ^a^	0.23 ± 0.02 ^a^	0.21 ± 0.02 ^a^	0.23 ± 0.03 ^a^	0.21 ± 0.02 ^a^	0.20 ± 0.00 ^a^
C20:4 n-6	1.36 ± 0.06 ^c^	1.36 ± 0.11 ^c^	0.99 ± 0.09 ^b^	0.91 ± 0.06 ^b^	1.52 ± 0.11 ^c^	1.39 ± 0.16 ^c^	0.83 ± 0.09 ^ab^	0.58 ± 0.03 ^a^
C18:3 n-3	0.71 ± 0.07 ^ab^	0.61 ± 0.04 ^a^	0.85 ± 0.02 ^b^	0.68 ± 0.05 ^ab^	1.05 ± 0.13 ^c^	0.71 ± 0.04 ^ab^	0.85 ± 0.03 ^b^	0.65 ± 0.07 ^a^
C18:4 n-3	1.33 ± 0.03 ^d^	1.23 ± 0.05 ^d^	0.92 ± 0.01 ^c^	0.84 ± 0.09 ^bc^	1.35 ± 0.18 ^d^	1.32 ± 0.10 ^d^	0.69 ± 0.01 ^ab^	0.60 ± 0.01 ^a^
C20:4 n-3	0.51 ± 0.05 ^b^	0.57 ± 0.05 ^b^	0.38 ± 0.03 ^a^	0.33 ± 0.03 ^a^	0.58 ± 0.05 ^b^	0.57 ± 0.05 ^b^	0.32 ± 0.02 ^a^	0.31 ± 0.03 ^a^
C20:5 n-3 (EPA)	9.29 ± 0.29 ^d^	8.32 ± 0.32 ^c^	4.02 ± 0.34 ^b^	3.51 ± 0.23 ^ab^	9.47 ± 0.26 ^de^	10.02 ± 0.19 ^e^	3.15 ± 0.26 ^a^	3.25 ± 0.04 ^a^
C22:5 n-3	2.22 ± 0.12 ^c^	2.19 ± 0.18 ^c^	1.01 ± 0.09 ^b^	1.23 ± 0.06 ^b^	1.92 ± 0.20 ^c^	2.17 ± 0.10 ^c^	0.66 ± 0.03 ^a^	0.64 ± 0.02 ^a^
C22:6 n-3 (DHA)	9.95 ± 0.90 ^c^	10.84 ± 1.04 ^cd^	4.89 ± 0.36 ^b^	3.86 ± 0.34 ^ab^	11.88 ± 0.75 ^d^	10.90 ± 0.21 ^cd^	2.55 ± 0.11 ^a^	2.36 ± 0.12 ^a^
Σ PUFA	27.22 ± 0.96 ^cd^	27.03 ± 1.12 ^c^	14.54 ± 0.54 ^b^	13.01 ± 0.44 ^b^	29.61 ± 0.87 ^d^	28.86 ± 0.36 ^cd^	10.35 ± 0.30 ^a^	9.66 ± 0.15 ^a^
Lipid quality indices								
n-6/n-3 PUFA	0.13	0.14	0.20	0.25	0.13	0.12	0.26	0.24
AI	0.67	0.67	1.09	1.12	0.67	0.68	0.96	0.96
TI	0.35	0.36	0.73	0.80	0.33	0.33	0.83	0.84
HH	1.82	1.78	1.13	1.10	1.88	1.88	1.15	1.13
FLQ	19.23	19.17	8.92	7.37	21.35	20.92	5.70	5.61

Results are reported as mean ± standard deviation (*n* = 3). Different superscript letters in each row mean significantly different values (*p* < 0.05). SFA: saturated FA; MUFA: monounsaturated FA; PUFA: polyunsaturated FA; AI: atherogenic index; TI: thrombogenic index; HH: hypocholesterolemic/hypercholesterolemic Index; FLQ: fish lipid quality index; n.d.: not detected.

## Data Availability

Data available on request.
